# Editorial Notes

**Published:** 1924-10

**Authors:** 


### Editorial Notes.

---

**The University accepts our Society's Library.** THE Medical Library, of which the Medico-Chirurgical Society is justly proud, was offered to and has been accepted by the Council of the University on conditions which preserve all the privileges of existing members and which are to be extended to all future members of the Society. The Council of the University, however, has shown their appreciation of this gift by providing a noble library with ample accommodation for its future expansion, as well as a large room for current periodicals and, furthermore, a room set aside for the use of the Society's members. We have no reason to doubt that the University, if not now, at any rate in the long run, will give to our Society as much if not more than they have received, and that our members will benefit very greatly from the transaction.

The Society's Journal has been a potent factor in the building up of this Library in the past, and should be no less so in its further development. The interests subserved by the Medical Faculty of the University and by this Journal are now more closely interwoven, and as a consequence we trust the sphere of our Journal's utility will be enhanced and widened.

We rejoice that Mr. L. M. Griffiths lived to know of this happy consummation of his great work for the Society, its Library and its Journal.

**The Colston Research Society.**

A GRANT from the Colston Research Society has made it possible to publish the clinical and pathological research on Auricular Failure by Drs. Carey Coombs and Herapath, and reproduce many of their original illustrations. It is not our purpose here to touch on the value of this contribution, though representing results of scientific investigation which advances our knowledge and solves some of the inherent problems of this aspect of cardiology, but we do feel that the Colston Research Society has earned the thanks of Medical Science for the encouragement and assistance afforded to such research and to our own Society by enabling us to bring the results before our readers in a complete form.

